# Sociodemographic characteristics and predictive factors of attrition: comparison in two final waves of a birth cohort study in Ecuador

**DOI:** 10.3389/frph.2025.1605182

**Published:** 2025-11-13

**Authors:** Nataly Cadena, Alexis J. Handal, Fabián Muñoz, Fadya Orozco

**Affiliations:** 1 Centro de Transferencia de Tecnología, Universidad San Francisco de Quito USFQ, Quito, Ecuador; 2 Department of Epidemiology, University of Michigan School of Public Health, Ann Arbor, MI, United States; 3 Visor Análisis Estadístico Cia. Ltda., Quito, Ecuador

**Keywords:** attrition analysis, birth cohort, drop out, low and mid income countries, Ecuador (country), sociodemographic factors, participant retention

## Abstract

**Background:**

Birth cohort studies are essential to investigate maternal and child health outcomes, yet they face persistent methodological challenges. A major concern is attrition, as participant loss over successive waves can compromise validity and introduce bias. These challenges are particularly acute in low- and middle-income countries, where socioeconomic inequalities and structural barriers further exacerbate participant loss and complicate long-term follow-up.

**Objective:**

This paper compares attrition between participants who remained and those who dropped out of the birth cohort study, SEMILLA. We analyze reasons for drop out, and the sociodemographic characteristics and predictive factors associated with attrition.

**Material and methods:**

Recruitment occurred over 30 months. Events such as the COVID-19 pandemic and social conflicts between 2019 and 2022 affected the final follow-up. The baseline sample included 409 pregnant women, divided into two Final Waves (FW): FW1 completed participation up to the baby's 12 months (*n* = 115), and FW2 up to 18 months (*n* = 294). Dropouts were identified by miscarriage, loss to follow-up, voluntary withdrawal, or protocol non-compliance. Baseline variables included ethnicity, years of schooling, maternal occupational activity, and per capita income. Attrition was calculated for each criterion overall and by Final Wave. Fisher's Exact Test, Pearson's chi-square, and Wilcoxon rank-sum tested differences between participants and dropouts. Logistic regression identified predictors of attrition in each Final Wave. All analyses were conducted with 95% confidence.

**Results:**

Of 409 participants, 94 dropped out: 19 in FW1 and 75 in FW2. The main reasons were protocol non-compliance (54%), voluntary withdrawal (21%), miscarriage (13%), and loss to follow-up (12%). In FW1, younger age was associated with attrition (*p* = 0.031), while in FW2, Mestiza ethnicity (*p* = 0.037) and lower income (*p* = 0.014) were significant. Logistic regression showed that older maternal age (OR = 0.87, *p* = 0.026) and higher income (OR = 0.99, *p* = 0.034) predicted lower attrition.

**Conclusion:**

Dropouts increased with longer follow-up, mainly due to time constraints. Age and income disparities significantly predicted continued participation. In contexts with socioeconomic challenges, these factors also affected protocol compliance. Findings underscore the importance of addressing socioeconomic determinants to strengthen the validity and sustainability of longitudinal studies in similar settings.

## Introduction

The implementation of community-based cohort studies during pregnancy, with the objective of following the population longitudinally, is essential to prospective studies and achieving answers to crucial questions about a variety of exposures that influence the health outcomes of the mother‒child binomial (1–3). However, these studies face multiple challenges, primarily the progressive decrease in the size of the initial sample during the follow-up waves, mainly due to loss to follow-up by study participants. A greater number of follow-up waves in studies tends to increase dropout rates (4–7). This methodological challenge may impact the results as a potential source of bias (8, 9).

A wide range of individual, family, social and demographic factors influence the decision to drop out of a birth cohort study. The literature shows that the implementation of this type of study in low- and middle-income countries, such as those in Latin America, faces greater challenges related to the complex political, economic and social context in which these studies are developed (1–3). In these countries, the percentages of disadvantaged and vulnerable populations with less education, lower income, lack of employment, and lack of social support, and of individuals who are ethnic minorities are high. These populations tend to be less likely to enroll in research studies and, in turn, have a greater probability of dropping out over time (1, 4, 10, 11). Furthermore, in these social contexts, women have a lower level of education, a lower socioeconomic status, and limited access to support networks; moreover, they are likely to be young mothers and have less autonomy. These characteristics reduce the chances that they will continue to participate in prenatal longitudinal research studies (12, 13).

In addition to the characteristics of the participants, there are factors corresponding to the study design that may increase dropout. These include repeated evaluations, long study durations, and short intervals between each follow-up wave, creating time, emotional, and social burdens for the participants (14, 15). Considering the aforementioned population and methodological characteristics, birth cohort studies often occur with complex structural determinants and a series of challenges to balance the technical design, methodological rigor and, not least, financial investment. In some cases, it becomes necessary to modify protocols to effectively and successfully complete the study (14, 15). This approach may include adjustments to the sample size, the number of waves for data collection and the total duration between the first and final waves (7). However, in the literature, information on attrition in these contexts, in which challenges involving the study design and the sociodemographic characteristics of the participants or in which identifying differences between those who remain in the study vs. those who decide to abandon it are taken into account, is limited (14–16). Notably, such characteristics are useful predictors of possible abandonment (6, 7, 9, 17, 18). On the basis of this knowledge deficit, this paper compares attrition between participants who remained and who dropped out—analyzing reasons, sociodemographic characteristics and predictive factors—in two final waves of birth cohort study, SEMILLA.

## Materials & methods

### Sample

The SEMILLA (Study of Environmental Exposure of Mothers and Infants Impacted by Large-Scale Agriculture) research project is a community-based birth cohort study implemented in Ecuador from August 2018 to the present in the Cayambe-Pedro Moncayo area. SEMILLA seeks to understand the associations between environmental exposure to pesticides, mainly the fungicide Mancozeb and its main metabolite ethylene thiourea (ETU), and the neurobehavioral development of infants according to maternal occupational activity (19).^1^

The study area was chosen because it contains a large proportion of 54% of the country's flower industry (20), of which the female workforce represents approximately 60% (21, 22). The total population of the area is 105,267 inhabitants, most of whom self-identify as Mestizo (62.5%) with the rest self-identifying as Indigenous (35.5%) or White (2%). In addition, the area has 30,040 households, with an average size of 3.5 members, although in some households, the number of members may exceed five (21, 22).

Inclusion criteria for the cohort included: being 18 years of age or older, between 8 and 20 weeks of gestation at enrollment, residing in the study area (Cayambe and Pedro-Moncayo cantons) for at least one continuous year prior to recruitment, and planning to remain in the area for at least one year after delivery. Based on these criteria, a total of 409 pregnant women were enrolled in the SEMILLA cohort. Detailed description of the study population, inclusion and exclusion criteria, and recruitment procedures are described elsewhere (19, see text footnote 1).

According to the original protocol, participants were expected to complete a baseline interview and up to 11 follow-up waves through pregnancy and until the child reached 18 months of age, with study completion planned for July 2022. However, the recruitment process lasted 30 months, from October 2019 to April 2022, due to periods of stoppage of activities in the field caused by events such as the COVID-19 pandemic and social conflicts in the country, which are currently still present (13). For this reason, it was necessary to modify the original protocol, extending the total duration of study implementation and fieldwork until October 2023. This modification involved a review of the follow-up time and the intervals between the interviews of each wave (from 11 to 9). Thus, the women recruited as of October 1, 2021, were able to complete their participation with at least the 12-month-old follow-up of their baby. In turn, the intervals of the interviews were adjusted to a quarterly format. For this reason, SEMILLA has two final waves with different completion times. The first final wave (FW1) included those mother‒child pairs whose participation period ranged from baseline to the infant's 12 month of age, and the second final wave (FW2) included those whose participation period ranged from baseline to the infant's 18 month of age.

Ethical approval was obtained by the Human Research Review Committee (HRRC) of the University of New Mexico (17–425; approved on 18 January 2018), the Health Sciences and Behavioral Sciences Institutional Review Board (IRB-HSBS) of the University of Michigan (HUM00138211, approved 8 August 2019), and the Human Research Ethics Committee of the Universidad San Francisco de Quito (CEISH ID 2017-177IN; approved 8 February 2018). Additionally, protocol approval was obtained by the Ecuadorian Ministry of Public Health National Health Intelligence Directorate (MSP—DIS; approved 14 August 2018), and the District Health Directorate 17D10 Cayambe, Pedro Moncayo, of the Ministry of Public Health, authorized the implementation of the study in the study region.

### Data collection procedures and measures

The study protocol is described in detail elsewhere (19), and we summarize it here. The data collection from the initial moment (baseline) and in each follow-up wave was carried out using various instruments, including the main survey, which covered sociodemographic, economic, maternal occupational activity, as well as other aspects related to social and health risk factors. The duration of this survey was approximately 1 h and 30 min. In addition, cognitive and psychometric scales were applied as well as health assessments of the pregnant participant. During the pregnancy follow-up, blood, urine, and hair and toenail samples were collected (19, see text footnote 1).

Within two weeks of birth, a venous blood sample was collected from the baby, and subsequently, nutritional and anthropometric evaluations, including a capillary blood sample for HemoCue, as well as a neurobehavioral developmental assessment test for each baby was performed. The biological samples were collected for 20 min. A maternal survey was also administered at each follow-up. In total, the duration of the follow-up visit of the participants was approximately 2 h (19, see text footnote 1).

**Figure 1:**
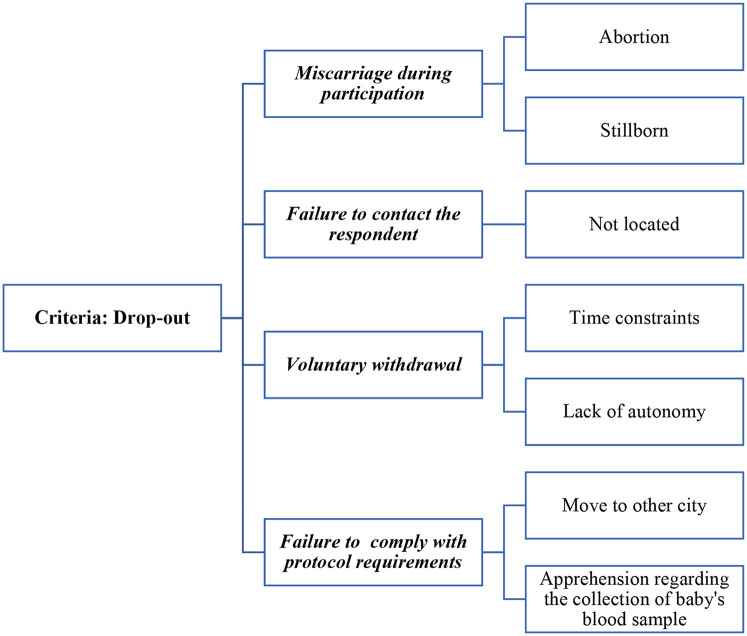
Drop-out criteria and reasons in SEMILLA study, Cayambe, Ecuador, 2020–2024.

The fulfillment of the follow-up activities, as well as the reasons reported by the participants for withdrawing from the study, were recorded in an Excel spreadsheet, called the “Tracking Planner” (23), where each participant had a numerical identifier, and individual information was entered weekly by the field team. This allowed the team to identify dropouts under the following criteria: (a) Miscarriage during participation; (b) Failure to contact the respondent; (c) Voluntary withdrawal; or (c) Failure to comply with the protocol requirements. Within each criterion, specific reasons were identified (See Figure 1).

### Explanatory variables

For the analysis of the comparisons between participants who remained and those who dropped out, as well as the predictors of attrition, variables collected during the baseline survey, related to the sociodemographic characteristics of the participants who remained in the study and those who subsequently dropped out, were selected. Among them, the following were included: (1) Ethnic self-identification, dichotomized as “Mestiza” or “ethnic minority”, the latter comprising the Indigenous (*n* = 88), Afro-Ecuadorian (*n* = 3) and White (*n* = 4) categories; (2) Marital status, which included the categories “married/partnered,” “separated/divorced/widowed,” and “single”; (3) Maternal occupational activity, categorized as “floricultural/agricultural work”, “non-agricultural work”, or “none”; (4) Years of schooling, considering the number of years reached by the participant in formal education in the country (23); (5) Number of people living in the household; (6) Mother's age, measured in years; (7) Gestational age, measured in weeks; and (8) Monthly per capita income was constructed by dividing the variable “monthly family income in dollars” by the “number of people who lived in the household” (23).

Additionally, two new variables were created: “Final Waves (FW)”, dichotomized, where 1 represented FW1 (baseline at 12 months of the baby) and 0 represented FW2 (baseline at 18 months of the baby). The dependent variable “status of participation” was also dichotomized, where 1 represented “dropped out” and 0 represented “remained”.

### Statistical analysis

The analyses were conducted with the statistical software STATA 18.0 and SAS v9.4.

To understand the attrition event, the percentages of participants who dropped out of the study were calculated for each criterion and reason, both in the study as a whole and for each Final Wave (FW) independently. Dropout proportions were calculated by dividing the number of dropouts by the total dropouts of the study as a whole, and by each FW.

The descriptive characteristics of participants who remained in the study and those who dropped out were obtained using relative frequencies for the qualitative variables, and means and standard deviations for quantitative variables. Sociodemographic differences between the two groups were evaluated using Fisher's Exact Test, Pearson's *χ*² test, and the Wilcoxon rank-sum test, according to FW.

For the analysis of the predictors (sociodemographic characteristics) of attrition, logistic regressions were performed, one for each FW. For all tests, the confidence level of the analyses was designated at 95%.

## Results

### Attrition criteria and reasons according to Final Waves

**Figure 2:**
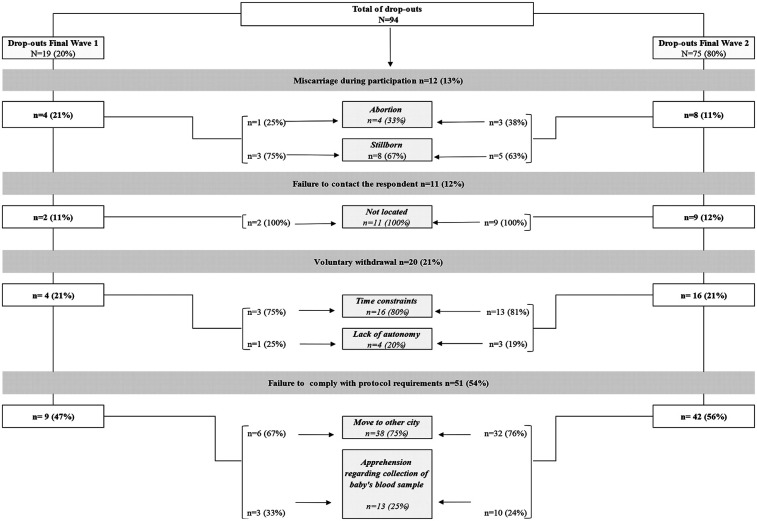
Criteria and reasons of those who dropped out according to Final Waves. SEMILLA study, Cayambe, Ecuador 2020–2024.

The total sample consisted of 409 participants, of which 115 belonged to FW1 and 294 to FW2. The total number of dropouts in the entire study was 94. The criteria and reasons for attrition, from lowest to highest frequency, were as follows: (1) 51 participants (54%) failed to comply with the protocol requirements, mainly because they moved to another city (*n* = 38, 75%), whereas apprehension regarding the collection of the baby's blood sample accounted for the remaining 25% (*n* = 13); (2) 20 participants (21%) voluntarily decided to withdraw from the study, with the main reason being time constraints (*n* = 16, 80%), rather than a lack of autonomy (*n* = 4, 20%); (3) 12 participants (13%) miscarried during participation, mostly due to stillbirth (67%) and, to a lesser extent, abortion (*n* = 4, 33%); and (4) 11 participants (12%) were unable to be contacted, as they could not be located (see Figure 2).

For FW1, the total number of dropouts was 19, and for FW2, it was 75. The main criteria for attrition were as follows: the participant failed to comply with the protocol requirements (47% in FW1% and 56% in FW2), mainly due to moving to another city (67% in FW1% and 76% in FW2), or the participant voluntarily decided to withdraw from the study (21% in both FW1 and FW2) due to time constraints (75% in FW1% and 81% in FW2). In addition, those who miscarried were predominantly in FW1 (21% vs. 11% in FW2). Finally, participants who were unable to be contacted accounted for 12% in FW2% and 11% in FW1, as they could not be located (see Figure 2).

### Sociodemographic differences between participants who remained and those who dropped out, according to Final Waves

From baseline to 12 months of age, corresponding to FW1, a significant difference (*p* < 0.05) in the ages of the women who remained (*n* = 96) and those who dropped out (*n* = 19) was observed. The average age of the dropouts was lower, with an approximate age difference of 3 years less than the average age of those who remained (24 vs. 27 years, *p* = 0.031). No significant differences (*p* > 0.05) in terms of marital status, ethnicity, gestational age at enrollment, or years of formal education were identified. Although dropouts represented a greater proportion of women outside the labor sector (21.05% vs. 15.62%), this difference was not significant (see Table 1).

**Table 1:** Baseline sociodemographic differences between participants who remained and those who dropped out, according to Final Waves. SEMILLA study, Cayambe Ecuador, 2020–2024.

Sociodemographic characteristics	FW1: Baseline to 12 months of the baby			FW2: Baseline to 18 months of the baby		
	Who stayed	Who dropped out	*P*-value	Who stayed	Who dropped out	*P*-value
	*n* = 96	*n* = 19		*n* = 219	*n* = 75	
	*n* (%)	*n* (%)		*n* (%)	*n* (%)	
Ethnic self-identification						
Mestiza	74 (77.08)	15 (78.95)	0.563	161 (73.52)	64 (85.33)	0.037*
Ethnic minority	22 (22.92)	4 (21.05)		58 (26.48)	11 (14.67)	
Marital status						
Married/united	79 (82.29)	14 (73.68)	0.594	168 (76.71)	55 (73.33)	0.832
Separated/divorced/widowed	2 (2.08)	1 (5.26)		7 (3.20)	3 (4.00)	
Single	15 (15.62)	4 (21.05)		44 (20.09)	17 (22.67)	
Maternal Occupational Activity						
Floriculture/agriculture	36 (37.50)	6 (31.58)	0.804	56 (25.57)	13 (17.33)	0.151
Non-agricultural work	45 (46.96)	9 (47.37)		73 (33.33)	22 (29.33)	
None	15 (15.62)	4 (21.05)		90 (41.10)	40 (53.33)	
	Mean ± Sd	Mean ± Sd		Mean ± Sd		Mean ± Sd
Number of people living in the household	3.59 ± 1.52	3.68 ± 2.08	0.720	4.10 ± 2.28	4.32 ± 1.89	0.180
Gestational age, measured in weeks	15.54 ± 2.72	16 ± 2.68	0.442	15.26 ± 3.37	14.81 ± 3.14	0.272
Mother's age	27.35 ± 5.69	24.42 ± 4.50	0.031*	27.53 ± 6.03	26.84 ± 5.62	0.380
Years of schooling	12.31 ± 3.95	11.55 ± 3.67	0.403	12.10 ± 3.56	12.53 ± 3.66	0.428
Monthly per capita income (USD)	230.22 ± 118.55	181.80 ± 82.46	0.085	168.96 ± 123.99	132.54 ± 93.14	0.014*

*p < 0.05, statistically significant.

From baseline to 18 months of age, corresponding to FW2, significant differences (*p* < 0.05) were observed in the characteristics of the women who remained (*n* = 219) and those who dropped out (*n* = 75). With respect to ethnic self-identification, the highest proportion of dropouts were Mestiza (85.33% vs. 73.52% of those who remained, *p* = 0.037). Similarly, the dropouts had a lower monthly per capita income, with an approximate difference of 36.42 USD less than that of those who remained (132.54 USD vs. 168.96 USD, *p* = 0.014) (see Table 1).

Finally, with respect to the analyzed predictors, the odds ratios (OR) revealed that, as the mother's age increased, the participants were less likely to drop out during FW1 (baseline period to 12 months of age) with an OR = 0.87 (95% CI 0.78–0.98, *p* = 0.026). Similarly, a higher monthly per capita income was associated with a lower probability of dropping out, especially during FW2 (baseline period to 18 months of age), with an OR = 0.99 (95% CI 0.96–0.99, *p* = 0.034) (see Table 2).

**Table 2:** Predictive factors of attrition according to Final Waves. SEMILLA study, Cayambe, Ecuador, 2020–2024.

Sociodemographic characteristics	FW1: Baseline to 12 months of the baby		FW2: Baseline to 18 months of the baby	
	*n* = 115		*n* = 294	
	OR (95% CI)	*p*-value	OR (95% CI)	*p*-value
Ethnic self-identification				
Mestiza	1.31 (0.36–4.66)	0.676	1.93 (0.92–4.04)	0.080
Ethnic minority	Reference group		Reference group	
Marital status				
Married/united	0.83 (0.21–3.25)	0.787	1.22 (0.59–2.49)	0.593
Separated/divorced/widowed	2.60 (0.11–61.72)	0.553	1.98 (0.38–10.36)	0.417
Single	Reference group		Reference group	
Maternal Occupational Activity				
Floriculture/agriculture	1.03 (0.29–3.61)	0.995	0.93 (0.41–2.10)	0.857
Non-agricultural work	Reference group		Reference group	
None	1.00 (0.29–3.61)	0.962	1.27 (0.66–2.45)	0.475
Number of people living in the household	0.91 (0.63–1.30)	0.605	0.99 (0.87–1.13)	0.939
Mother's age	0.87 (0.78–0.98)	0.026*	0.98 (0.93–1.03)	0.519
Years of schooling	0.98 (0.85–1.13)	0.827	1.05 (0.97–1.15)	0.236
Monthly per capita income (USD)	0.99 (0.99–1.00)	0.154	0.99 (0.96–0.99)	0.034*

*p < 0.05, statistically significant.

## Discussion

The findings from this analysis highlight important reasons for attrition for participants in a community-based birth cohort study, SEMILLA. During Final Waves 1 and 2, the main criteria and reasons for attrition were as follows: (1) failure to comply with protocol requirements, mainly due to moving to another city with a smaller proportion noting apprehension about blood collection from their baby; and (2) voluntarily deciding to withdraw from the study, mainly due to time constraints. With respect to the first reason, in Ecuador, post COVID-19 pandemic, internal migration affected the labor market, increasing uncertainty and family economic conflicts, which prompted migration in search of other employment opportunities (24). According to the World Bank (2021), 77% of families in low- and middle-income countries lost their jobs during this time (7, 25). In Ecuador, an estimated 1.5 million people fell into poverty, forcing many to migrate in search of work in other cities and regions (24). To a lesser degree, in this study, apprehension regarding the collection of the baby's blood sample, was noted as a reason for failure to comply to study protocol in 25% of those who dropped out. This is an aspect that is mentioned in the literature because the most vulnerable research participants tend to experience greater anxiety around procedures for obtaining biological samples from their children. This fear may be linked to widespread mistrust in contexts of vulnerability and social insecurity. For example, a prospective pediatric cohort study on typhoid surveillance in Vellore, South India, revealed that mothers who perceive their children to be at risk in research studies are more likely to drop out. For these women, it may be easier to participate during pregnancy (26). Similarly, Daniels et al. (2006) reported that women in poverty are often particularly concerned with the collection of physical or biological data related to their offspring, especially during the neonatal period. Finally, Brumatti et al. (2013) reported that the participants in the Phime, Italy cohort study were concerned with the possible risks associated with the collection, long-term storage and future use of biological samples from their children, as well as exposure to long and stressful neurodevelopmental tests (25). This discomfort with the study procedures and activities contributes to the increase in loss to follow-up, especially among the most vulnerable women (27).

With respect to the decision to leave the study voluntarily, Kingston and Jagger (2017) have suggested that the follow-up methodology used in a cohort study plays an important role. For example, participation in studies that require a high volume of data collection with surveys that last more than an hour and with short intervals between each wave of follow-up can be an overwhelming time load for women with limited resources and multiple responsibilities (15, 33). Other authors, such as Goldstein et al. (2021), agree that the high burden of participation makes it difficult for women to balance their involvement in the study with their family and work activities. Additionally, the authors highlight that privacy concerns may also influence the women's decisions to withdraw from a research study especially during situations of economic and social vulnerability. Some participants may feel uncomfortable sharing personal information or fear the disclosure of sensitive data (27).

With respect to the sociodemographic characteristics of the participants, a significant difference was observed between the women who remained in the study and those who dropped out. In FW1, those who dropped out were younger; whereas in FW2, they were predominantly Mestiza and had a lower monthly per capita income. When considering the finding on age, in this context, retention in birth cohort studies has been found to increase among older participants (24). In fact, in India, the participation rates were 90% among older women and 68% among younger women (26). Additionally, Sindhu, Srinivasan et al. (2019) noted that the factors contributing to high attrition rates among young mothers are low literacy levels, low socioeconomic strata, and ethnic minority status (10). Additionally, the literature indicates that in Latin American contexts, young women tend to lack autonomy, which may significantly affect their participation in birth cohort studies. This lack of autonomy can be understood from various perspectives, such as the control exercised by the partner, or the family or the social norms that limit their ability to make personal decisions. In various sociocultural contexts, the interruption of women's participation in birth cohort studies can be influenced by the opinions of the couple, especially if they consider such studies to be uncomfortable or waste time. This is particularly evident in traditional family structures, where women must assume domestic or caring responsibilities, making it difficult to balance those obligations with the demands of study (25, 27, 28).

With respect to ethnicity, in the present study, most women self-identified as Mestiza, and this may have contributed to the higher percentage of dropouts in this category for FW2. Our findings noted more attrition in FW2 for those with lower monthly per capita income. Young, Powers and Bell (29) note that in community cohort studies conducted in countries with characteristics similar to those of Ecuador, women with better economic conditions are more likely to continue in a prospective study because it may be easier for them to cover the costs associated with the time invested. They may also face fewer social, cultural and educational barriers that would prevent them from fully understanding the benefits of participating in the research (1, 23).

Concerning the findings on the predictors of attrition, significant disparities were observed between the women who remained in the study and those who dropped out, the latter being those who seem to be in worse socioeconomic conditions, which may have increased their low autonomy and greater vulnerability. Participants with lower incomes face greater economic difficulties, making them prioritize the need to work or attend to financial problems. In addition, they tend to have limited access to resources, so they do not perceive immediate direct benefits from participating in a prospective study, such as financial compensation (30–32). On the other hand, women with low financial resources also face barriers to support domestic and caregiving responsibilities, which makes it difficult for them to dedicate time to participate in these types of cohort studies (25, 27).

Among the main limitations of the study are additional factors that could have contributed to the observed attrition that were not considered, such as having timely information on the perceptions of the participants about the benefits of the study. Similarly, having more participants in FW2 than in FW1 could have generated an unbalanced representation in the analysis of the Final Waves, which could have made comparisons related to the time of participation difficult. No less important are the contextual factors that affected the development of the study, which may have influenced attrition. Despite these limitations, the present study included various sociodemographic variables, which allowed for a detailed analysis of the factors that could influence study attrition. Additionally, the fact that the study was completed through two Final Waves provided the opportunity to compare different follow-up periods and analyze how the dropout factors may vary over time and between groups.

In sum, factors such as younger age and having a lower income seem to be relevant in both high-income countries and low- and middle- income countries, although studies in which the characteristics of dropouts and the reasons for them in low- and middle-income countries remain limited (30–32). The sociodemographic characteristics of the women who dropped out of the SEMILLA study may have potential implications for the study's results and should be considered in future analyses, as existing literature suggests that factors such as maternal age and income can influence the neurobehavioral development of children (30–32).

## Conclusions

Attrition analysis in the SEMILLA study showed that dropouts increased with longer follow-up duration. The main reason was protocol non-compliance, often linked to time constraints and competing demands faced by women in vulnerable socioeconomic conditions. Age, income, and ethnicity emerged as important predictors of continued participation, highlighting structural inequities that affect retention in longitudinal studies.

These findings emphasize that attrition is not only a procedural challenge but also a reflection of broader social determinants. Future cohort studies in similar contexts should incorporate strategies to reduce participant burden, such as flexible scheduling, simplified follow-up protocols, and hybrid data collection modalities, and strengthen engagement mechanisms to minimize loss to follow-up. Providing targeted support for younger, lower-income, and ethnically marginalized women could help ensure greater inclusivity and validity in long-term maternal child health.

## Data Availability

Once the SEMILLA data have been fully analyzed and the main findings published, data sharing will be considered for qualified researchers. Given that our study population is vulnerable, particular care must be taken to protect participants' privacy. Data sharing will only occur under a formal agreement, ensuring compliance with ethical guidelines and institutional regulations. Such agreements will require users to: (1) utilize the data strictly for predefined and approved research purposes, (2) obtain prior approval from their Institutional Review Board (IRB), (3) ensure secure storage and handling of the data, (4) acknowledge and cite the SEMILLA study and its principal investigators in all outputs, and (5) delete or return the data upon completion of the agreed analyses. Requests for access will be reviewed on a case-by-case basis by the principal investigators.

## Footnotes

1 Orozco F, Montenegro S, Muñoz F, Handal AJ. Recruitment process for studying occupational and environmental chemical exposures in a birth cohort study in Ecuador: challenges dealing with a stratified sample. *BMC Pregnancy and Childbirth*.
